# Tunable lipid-coated nanoporous silver sheet for characterization of protein-membrane interactions by surface-enhanced Raman scattering (SERS)

**DOI:** 10.1007/s00216-023-04701-y

**Published:** 2023-04-21

**Authors:** Hongni Zhu, Jianing Zhang, Xin Dai, Vince St. Dollente Mesias, Huanyu Chi, Congcheng Wang, Chi Shun Yeung, Qing Chen, Wei Liu, Jinqing Huang

**Affiliations:** 1 HKUST-Shenzhen Research Institute, No. 9 Yuexing First RD, Hi-Tech Park, Nanshan, , Shenzhen, 518057 China; 2 Department of Chemistry, The Hong Kong University of Science and Technology, Clear Water Bay, Kowloon, Hong Kong, China; 3 Department of Chemistry, The University of Hong Kong, Pokfulam Road, Hong Kong, China; 4 Department of Mechanical and Aerospace Engineering, The Hong Kong University of Science and Technology, Clear Water Bay, Kowloon, Hong Kong, China; 5 Department of Civil & Environmental Engineering, The Hong Kong Polytechnic University, Hong Kong, China

**Keywords:** SERS, Protein-membrane interactions, Hydrophilic effect, Hydrophobic effect

## Abstract

Membrane environments affect protein structures and functions through protein-membrane interactions in a wide range of important biological processes. To better study the effects from the lipid’s hydrophilic and hydrophobic interaction with protein on different membrane regions, we developed the lipid-coated nanoporous silver sheets to provide tunable supported lipid monolayer/bilayer environments for in situ surface-enhanced Raman vibrational spectroscopy (SERS) characterizations. Under the controllable surface pressure, lipid monolayer/bilayer was coated along the microscopic curved surface of nanoporous silver sheets to serve as a cell membrane mimic as well as a barrier to avoid protein denaturation while empowering the high SERS enhancements from the underlying metallic bases allowing detection sensitivity at low physiological concentrations. Moreover, we fine-tuned the lipid packing density and controlled the orientation of the deposited lipid bilayers and monolayers to directly monitor the protein structures upon interactions with various membrane parts/positions. Our results indicate that lysozyme adopted the α-helical structure in both hydrophilic and hydrophobic interaction with lipid membrane. Interestingly, alpha-synuclein folded into the α-helical structure on the negatively charged lipid heads, whereas the hydrophobic lipid tails induced the β-sheet structural conversion of alpha-synuclein originated from its unstructured monomers. These direct observations on protein hydrophilic and hydrophobic interaction with lipid membrane might provide profound insights into the formation of the β-sheet-containing alpha-synuclein oligomers for further membrane disruptions and amyloid genesis associated with Parkinson’s disease. Hence, with the controllability and tunability of lipid environments, our platform holds great promise for more general applications in investigating the influences from membranes and the correlative structures of proteins under both hydrophilic and hydrophobic effects.

**Graphical Abstract:**

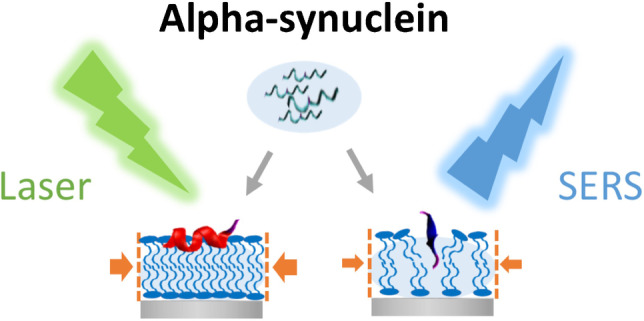

**Supplementary Information:**

The online version contains supplementary material available at 10.1007/s00216-023-04701-y.

## Introduction

Cell membranes host many significant biological processes via protein-lipid interaction. Lipid molecules are amphipathic that could impost both the hydrophilic effect via lipid heads and the hydrophobic effect with lipid tails. Membrane proteins bind to the polar lipid heads at the water/lipid interface mainly through electrostatic interactions. While, hydrophobic interactions dominate when the proteins start to insert into membranes, where the nonpolar lipid tails facilitate altering and stabilizing protein secondary structures via hydrophobic interaction [1]. Occasionally, the packing defects of lipid layers could expose the hydrophobic lipid tails to initiate the folding and insertion of hydrophobic protein residues associated with diseases. For example, it is reported that the interactions between alpha-synuclein and membranes promote its oligomerizations and fibril formations [2, 3], which are the hallmark of Parkinson’s disease. In particular, alpha-synuclein preferably binds to negatively charged membranes with highly curved membrane surfaces and lipid packing defects [4], where the membrane disruption levels depend on the accessibility of the lipid hydrophobic regions [5]. Even monomeric alpha-synuclein at micromolar concentrations could damage lipid vesicles [6]. However, the molecular mechanisms underlying the interactions between the distinct membrane regions and the disease-related structural transitions of the membrane-related proteins like alpha-synuclein remain unclear. Hence, it is of great importance to construct well-defined yet controllable lipid-membrane environments with sensitive characterizing techniques to investigate various protein-membrane interactions.

Surface-enhanced Raman spectroscopy (SERS) is a powerful tool to study in situ protein-membrane interactions. Utilizing the localized surface plasmon resonances (LSPRs) in the metallic nanostructures, SERS substrates empower the detection of biomolecules in dilute aqueous solutions [7, 8] toward the single-molecule sensitivity [9, 10]. Moreover, it directly probes the intrinsic molecular vibrations to characterize protein structures in a label-free manner [11], which has been applied to uncover the secondary structures of amyloid proteins including beta-amyloid and islet amyloid polypeptides [12–17]. To provide membrane environments, the SERS substrates have been decorated in the form of lipid-coated nanoparticles [12, 18], nanoparticles on supported lipid layers [19, 20], and liposome-sandwiched nanoparticle-on-mirror substrates [21], serving as a versatile platform to analyze biomolecules. Nevertheless, most of the lipid-coated SERS substrates are deposited by vesicle fusions or covalent bond formations, which limit the capability to independently control specific membrane properties, in particular, lipid packing defects. Since these lipid attachments solely rely on the spontaneous interactions between lipid molecules and SERS substrates, it is less straightforward to fine-tune the lipid packing density and layer orientation to mimic different biological membrane conditions for systematic assessments. Alternatively, Langmuir–Blodgett (LB) technique can pack amphiphilic lipid molecules into an oriented monolayer at the air/water interface with precise control of the lipid lateral pressure and transfer it onto a solid substrate under the constant compressing pressure to ensure a high-quality lipid coating. It provides a convenient approach to manipulating the composition and the number of lipid layers on the substrate to accommodate various research needs [22]. Furthermore, these solid-supported lipid monolayer/bilayers have been integrated with in situ microscopy and spectroscopy techniques to investigate the secondary structures of proteins in membrane interactions, demonstrating superior advantages from both qualitative and quantitative perspectives [23]. Although a few studies involve SERS measurements, their applications are limited to material developments [24, 25]. The analytical power of combining the LB depositions of biological lipid monolayer/bilayers as membrane mimics toward the SERS characterizations of protein-membrane interaction has been scarce.

Here, we utilized the LB technique to construct a tunable lipid-coated nanoporous silver sheet for characterizing the protein secondary structures upon protein-membrane interactions under both hydrophilic and hydrophobic effects. The pressure-controlled LB transfer method ensures the homogenous and stable lipid layers coated along the curved surface of the underlying metallic nanostructures, providing high SERS enhancements while avoiding potential protein denaturation for sensitive SERS measurements. To mimic biological membrane conditions, we adjusted the lipid packing density via surface pressure of the deposited lipid bilayers and monolayers, which could directly monitor the protein structures upon the electrostatic interactions with lipid heads and the hydrophobic interactions with lipid tails, respectively. Two membrane-related proteins, lysozyme and alpha-synuclein, were investigated at different lipid packing conditions. In particular, alpha-synuclein demonstrated distinct structural features when interacting with the hydrophilic lipid heads and the hydrophobic lipid tails, which might be linked to its misfolded oligomers for membrane insertions and disruptions associated with the pathogenic mechanism of Parkinson’s disease. Therefore, with the lipid lateral pressure controllability of LB and the structural sensitivity of SERS, our platform could exploit the tunable lipid membrane environments to reveal the driving force of the protein secondary structural changes for significant protein-membrane interactions.

## Materials and methods

### Materials

Silver foil (Ag, 99.9%, 0.127 mm thick) was purchased from Alfa Aesar (Massachusetts, USA). Potassium chloride, sodium borohydride, sodium chloride, Tris base, hydrochloric acid, methanol, yeast extract, tryptone, kanamycin, isopropyl β-D-1-thiogalactopyranoside, and lysozyme were purchased from Sigma-Aldrich (St. Louis, MO). DPPG (99%) was purchased from Avanti Polar Lipids, Inc. (Alabaster, AL) and used as received. Chloroform and methanol (RCI Labscan Limited, Bangkok, Thailand) were used to dissolve lipids. Water (resistivity of 18.2 MΩ cm) for all experiments was supplied by the Milli-Q system.

### Expression and purification of alpha-synuclein

The plasmids of wild-type alpha-synuclein were transformed into *E. coli* BL21(DE3), and cells were spread on an LB agar plate supplemented with 25 µg/mL kanamycin and incubated at 37 °C overnight. After that, one single colony was inoculated into 10 mL LB medium supplemented with 25 µg/mL kanamycin and shaken at 37 °C overnight. 2 mL cell culture was transferred to 1 L LB with 25 µg/mL kanamycin. The culture was continued to shake and grow to around OD_600_ = 0.6 for about 5 h. 0.3 mM isopropyl β-D-1-thiogalactopyranoside (IPTG) was added for induction, and the culture was shaken at 37 °C for another 4 h at 250 rpm. The cells were then harvested by centrifugation for 15 min at 4200 rpm and 4 °C. The cell pellet was resuspended in 40 mL lysis buffer (25 mM Tris buffer, pH 8.0) and lysed by sonication. After centrifugation at 18,000 rpm for 30 min at 4 °C, the supernatant was boiled to remove the proteins from *E. coli*. The boiled supernatant was then centrifugation at 18,000 rpm for another 60 min at 4 °C, and the pellet was discarded. The supernatant was filtered and loaded onto HiPrep DEAE FF 16/10 column (GE Healthcare). A gradual sodium chloride gradient was applied to elute the target protein. After SDS-PAGE gel analysis, the fractions containing alpha-synuclein were desalted by HiPrep™ 26/10 Desalting column (GE Healthcare). The desalted solution was loaded onto HiPrep 16/60 Sephacryl S-100 column (GE Healthcare) for further purification (25 mM Tris, pH 8.0 as the running buffer). Fractions were analyzed by SDS-PAGE gel, and targeted protein was collected, concentrated, and stored at − 20 °C.

### Fabrication of nanoporous silver sheet

The nanoporous silver sheets were fabricated by the electrochemical method as previously reported [26]. The pure silver sheets (99.9%) were first washed with distilled water and then put into 1 M potassium chloride aqueous electrolyte for electrochemical reaction. After that, the metal surface turned from lustrous silver into dark yellow, which suggested that silver chloride was coated on the surface of silver sheets. The silver coating was then quickly placed into 0.1 M sodium borohydride solution and reduced to silver. The nanoporous silver sheets were acquired until visible bubbles no longer appeared in the solution. Finally, the sheets were washed with water and ethanol three times, respectively. They were dried and preserved in the bottle with air or inert gas.

### Preparation of lipid-coated nanoporous silver sheet

To prepare lipid-coated nanoporous silver sheets, the Langmuir–Blodgett method was used to prepare and transfer lipid monolayer (or bilayer) onto the nanoporous silver sheets. A 364 × 74 mm^2^ KSV trough (Biolin Scientific, Gothenburg, Sweden) was used to prepare the Langmuir monolayer lipid films, and the surface pressure of the films was measured by a Wilhelmy paper plate pressure sensor. Milli-Q water was used as the subphase in the trough. Thirty microliter of 1.0 mg/mL lipid solution was spread onto the water subphase by using a Hamilton micro-syringe. After the solvent evaporation for about 15 min, the film was compressed at a constant sweeping speed of 5 mm/min. The nanoporous silver sheet was stacked on the slide and attached to the KSV dipper. Before dipping, the lipid monolayer was compressed to the target surface pressures, and the slide was pulled up for monolayer coating at a constant dipping rate of 1 mm/min, while the surface pressure was maintained. To deposit lipid bilayers onto the nanoporous silver sheets, a second-round dipping was performed on the lipid-coated nanoporous silver sheet. Before dipping, the lipid monolayer was compressed to the target surface pressure, and the slide was pulled down for monolayer coating at a constant rate of 1 mm/min, while the surface pressure was maintained. The calculated transfer ratios are summarized in Table 1. The TR of the lipid monolayer transferred onto the nanoporous substrate is larger than 1 due to the microscopic curvature on the substrate surface. In the bilayer dipping using LB-LB method, the TR is lower than the first layer of dipping, implying fewer lipids were transferred due to the weaker intermolecular affinity. In general, at higher surface pressure more lipids were transferred onto the substrate, yet TR in all conditions suggest that all substrates were coated with a reasonable amount of lipids molecules to form membrane film, with less packing density compared to the film formed at the air–water interface.

**Table 1:** Parameters for lipid deposition

Surface pressure	Dipping rate	Compression rate	Transfer ratio
15 mN monolayer	1 mm/min	2 mm/min	1.12
15 mN bilayer			0.87
25 mN monolayer			1.21
25 mN bilayer			0.98
35 mN monolayer			1.32
35 mN bilayer			1.12

### Characterization of lipid-coated nanoporous silver sheet

The morphology of the nanoporous silver sheet before and after lipid treatment was observed by scanning electron microscopy (SEM, JSM-6390, JEOL, USA Inc.). The background spectra of bare nanoporous silver sheet and lipid-coated nanoporous silver sheet were obtained using an in-Via confocal Raman microscope (Renishaw, Gloucestershire, UK) with a 514.5 nm laser at 5 mW focusing by a 20 × microscope objective on the sample. The spectral acquisition time was 30 s. At least three repeated measurements were conducted at different spots of the samples. All spectra were plotted and analyzed after baseline correction by Renishaw WIRE (ver 4.0, UK).

### Protein detection on lipid-coated nanoporous silver sheet by Raman and SERS measurement

Lysozyme and alpha-synuclein were chosen to prepare 1 mM and 1 μM protein aqueous solutions. 1 mM protein solution was dropped on a glass slide with or without the lipid coating for spontaneous Raman measurements. The spontaneous Raman measurements were conducted on a confocal Raman microscope (in-Via, Renishaw, Gloucestershire, UK) using a 514.5 nm laser at 100 mW with a 20 × microscope objective to focus on the surface of the sample droplets. The acquisition time was 10 min with 3 repeats for accumulation. The spectra were analyzed after background subtraction, which was obtained under identical conditions without samples. For SERS measurements, 7 μL of 1 μM protein solution was dropped on the lipid-coated nanoporous silver sheet and incubated for 2 h. Using the confocal Raman microscope (in-Via, Renishaw, Gloucestershire, UK), SERS spectra were obtained with the 514.5 nm laser at the laser power of 5 mW and the 20 × microscope objective to focus on the sample substrates. The spectral collection time was 30 s with 3 repeats for accumulation. The SERS spectra were analyzed after baseline correction by Renishaw WIRE (ver 4.0, UK). All spectra were plotted using OriginPro 2022.

## Results and discussion

**Fig. 1:**
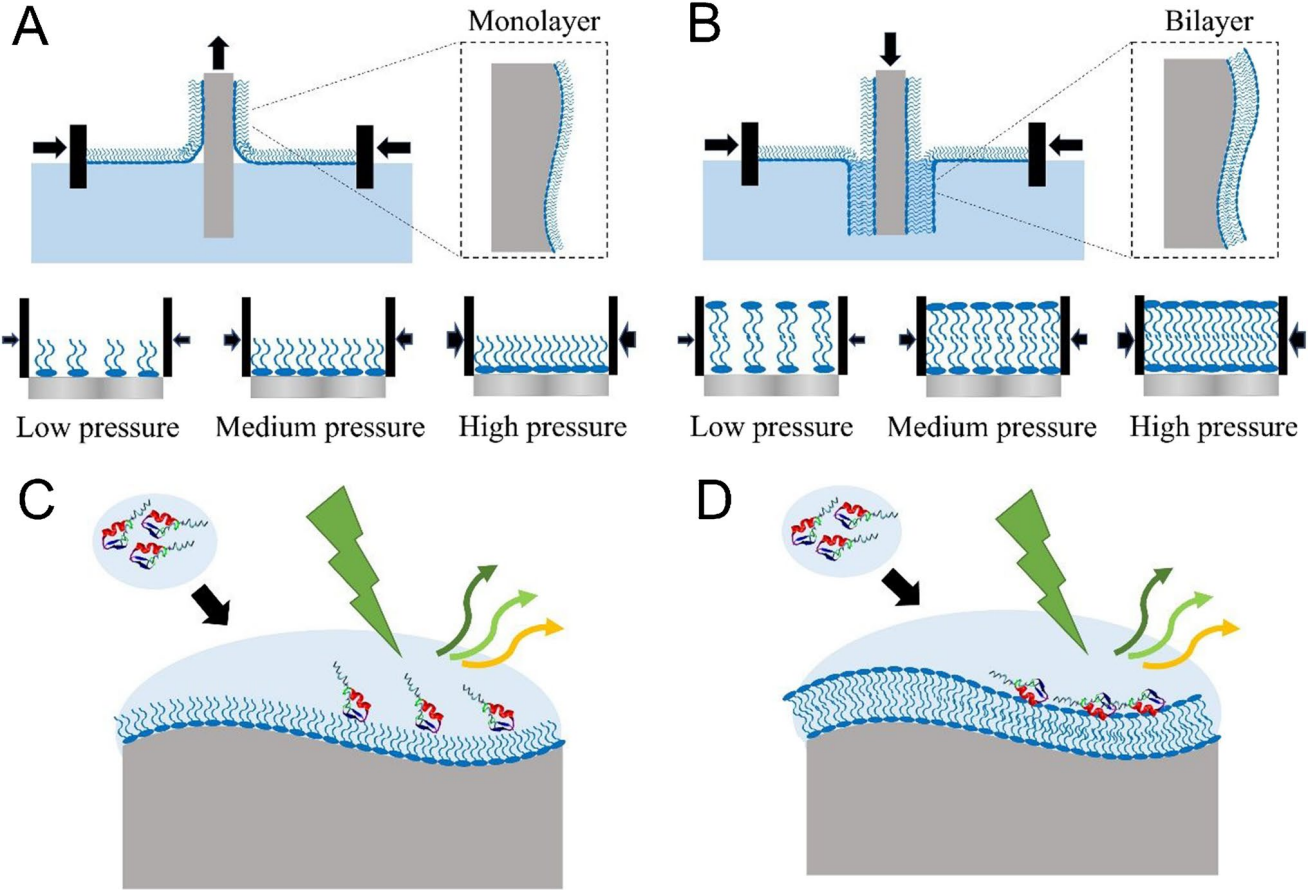
Preparations of the lipid-coated nanoporous silver sheets for the SERS characterizations of proteins in tunable lipid environments. Schematics of the Langmuir–Blodgett deposition of the lipid monolayer (**A**) and bilayer (**B**) at the controllable surface pressure onto the nanoporous silver sheets to provide tunable lipid environments. Illustrations of the SERS measurements of proteins upon the interaction with the lipid tails on the monolayer lipid-coated nanoporous silver sheet (**C**) and the lipid heads on the bilayer lipid-coated nanoporous silver sheet (**D**)

Figure 1A and 1B illustrate the preparation of the lipid-coated nanoporous silver (NPAg) substrate by the Langmuir–Blodgett transfer of the lipid monolayer(s) from the air–water interface onto the NPAg sheet. First, DPPG lipid molecules were compressed into a homogeneous monolayer at the air–water interface under target surface pressure. By slowly pulling the sheet up from the water phase into the air phase while maintaining the surface pressure of the lipid monolayer using two symmetric barriers, the hydrophilic lipid heads were coated along the curved surface of the NPAg sheet with the outward lipid tails to provide the hydrophobic lipid environments, as shown in Fig. 1A. By pushing the previous sheet down from the air phase into the water phase while maintaining the surface pressure of the lipid monolayer, the lipid bilayer was generated along the curved surface of the NPAg sheet, providing a hydrophilic environment as cell membrane mimic as shown in Fig. 1B. The surface pressure level of the lipid monolayer was adjusted to low (15 mN/m), medium (25 mN/m), and high (35 mN/m), corresponding to liquid phase, solid phase, and typical cell membrane pressure, respectively, which directly controlled the lipid packing density of the deposited lipid monolayer and bilayer. Contact angle measurements were conducted to examine the surface hydrophobicity of the monolayer and the bilayer lipid-coated NPAg sheets. Figure S1 presents the contact angles as 108° ± 1°, 112° ± 2°, and 119° ± 0° for the monolayer lipid-coated NPAg sheets under the surface pressure of 15 mN/m, 25 mN/m, and 35 mN/m, respectively, and 79° ± 0°, 75° ± 1°, and 66° ± 3° for the bilayer lipid-coated NPAg sheets under the surface pressure of 15 mN/m, 25 mN/m, and 35 mN/m, respectively. The contact angles larger than 90° indicate hydrophobic surfaces, which verify that the monolayers with outwards lipid tails were coated on NPAg sheets. The contact angles smaller than 90° suggest hydrophilic surfaces, owing to the lipid bilayers with outwards head groups coated on the NPAg sheets. Furthermore, the contact angles change with the surface pressures for lipid depositions, which prove the different surface hydrophobicities related to the different packing densities on the lipid-coated NPAg sheets. Hence, the proteins in the surrounding aqueous environment are allowed to interact with monolayer lipid hydrophobic tails and with bilayer lipid hydrophilic head groups. As illustrated in Fig. 1C and D, the membrane proteins were directly characterized upon interacting with different membrane environments on the lipid-coated NPAg sheets by SERS spectroscopy via the enhancement provided by the underlying metal substrates.

**Fig. 2:**
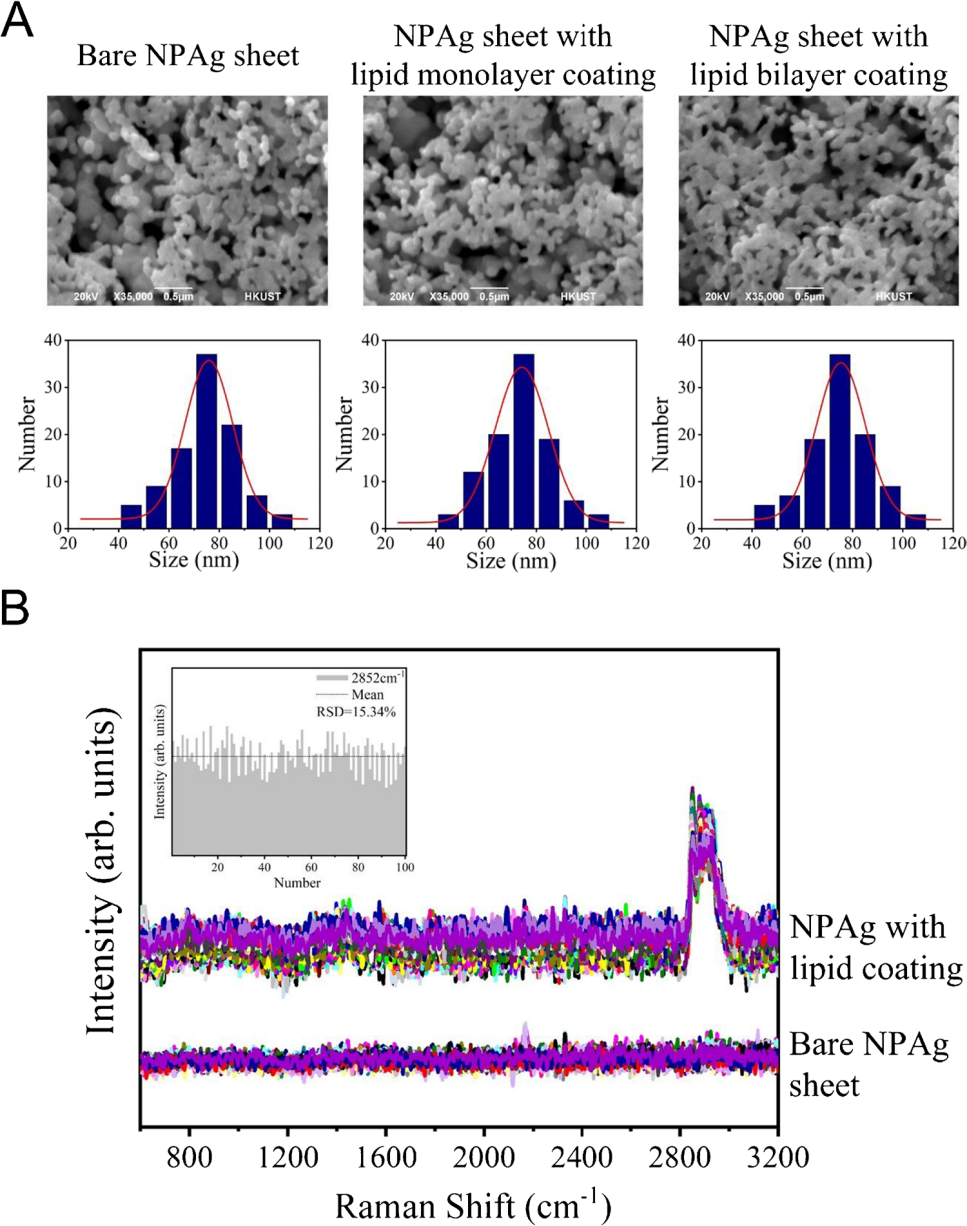
Characterizations of the lipid-coated NPAg sheets. **A** The SEM images of the bare NPAg sheet, the monolayer lipid-coated NPAg sheet, and the bilayer lipid-coated NPAg sheet, showing the porous morphology (top) and porous size distribution (bottom). **B** SERS spectra of the NPAg sheets before (bottom) and after (top) the lipid deposition. (SERS spectra acquisition: 5mW 514.5 nm laser, 20 × objective, 30 s) Inset: The histogram of the intensity of the lipid characteristic peak at 2852 cm^−1^ (mean = 200.19 in arb. units, RSD = 15.34%) across 100 SERS spectra acquired at different spots on the sheets

Figure 2A shows the surface morphologies of the bare NPAg sheet, the monolayer lipid-coated NPAg sheet, and the bilayer lipid-coated NPAg sheet, which all possess uniform structures with coral rock-like nanosilver-rods and abundant narrow pores. The statistical analysis of the pore size distribution from SEM images indicates the average size of 70–80 nm, which is further verified by the Brunauer–Emmett–Teller (BET) method based on adsorption isotherms of nonreactive nitrogen (Fig. S2). The morphological features of the NPAg sheet are preserved after the deposition of the lipid monolayer and the bilayer, providing abundant SERS hotspots with the enhancement factor of 10^6^ as reported in the previous study. It is worth notice that after the lipid coating, the size distribution is slightly smaller. This is due to the coating thickness on surface curvature, while more holes in underneath layers remain uncoated. Leveraging the porosity, the lipid-coated NPAg sheets not only support tunable and controllable lipid environments, but also enlarge the probe volume and lower the detection concentration for SERS measurements. In addition, Fig. S3 compares the Raman spectrum of the lipid monolayer coated on the glass slide and that of the lipid monolayer coated on the Ag substrate, showing similar peaks at around 2849 cm^−1^, 2874 cm^−1^, and 2928 cm^−1^ for the symmetric C-H stretching of the methylene, the antisymmetric C-H stretching of the methylene, and the symmetric C-H stretching of the terminal methyl groups, respectively. Based on previous studies [27], the DPPG head groups were squeezed down and attached to the metal substrate while exposing the vertically oriented lipid tails, which is further verified by the surface hydrophobicity in the contact angle test. To inspect the lipid coating coverage, the SERS spectra were acquired at 100 different spots of the NPAg sheets before (bottom) and after (top) the lipid deposition, as compared in Fig. 2B and Fig. S4. At different spots of the monolayer lipid-coated NPAg sheet, the 100 SERS spectra all demonstrate spectral features at around 2800–3000 cm^−1^, assigned to the C-H stretching of the DPPG phospholipid molecule [28], indicating the successful coating of the lipid monolayer/bilayer. Moreover, the histogram of the peak intensities at 2852 cm^−1^ across the 100 SERS spectra shows the relative standard deviation (RSD) as 15.34% in the inset of Fig. 2B, which is a satisfactory fluctuation (< 20%) for conventional SERS substrates considering the possible lipid coating inhomogeneity [29]. Furthermore, the overall peak intensities of the monolayer lipid-coated NPAg sheets and the bilayer lipid-coated NPAg sheets rise with the increasing surface pressures and the corresponding lipid packing densities. In particular, the intensity ratios of the symmetric and the antisymmetric C-H stretching modes (***I***_2928_/***I***_2850_) change from 1.80 and 2.63 to 3.26 for monolayers and from 1.64 and 2.96 to 4.23 for bilayers at the surface pressure of 15 mN/m, 25 mN/m, and 35 mN/m, respectively, which implies the different lateral packing orders on the lipid-coated NPAg sheets [30]. These observations verify the quality of lipid coating and the consistent SERS enhancements at different spots of the lipid-coated NPAg sheet, which lays the foundation for the following in situ characterizations of protein-membrane interactions.

**Fig. 3:**
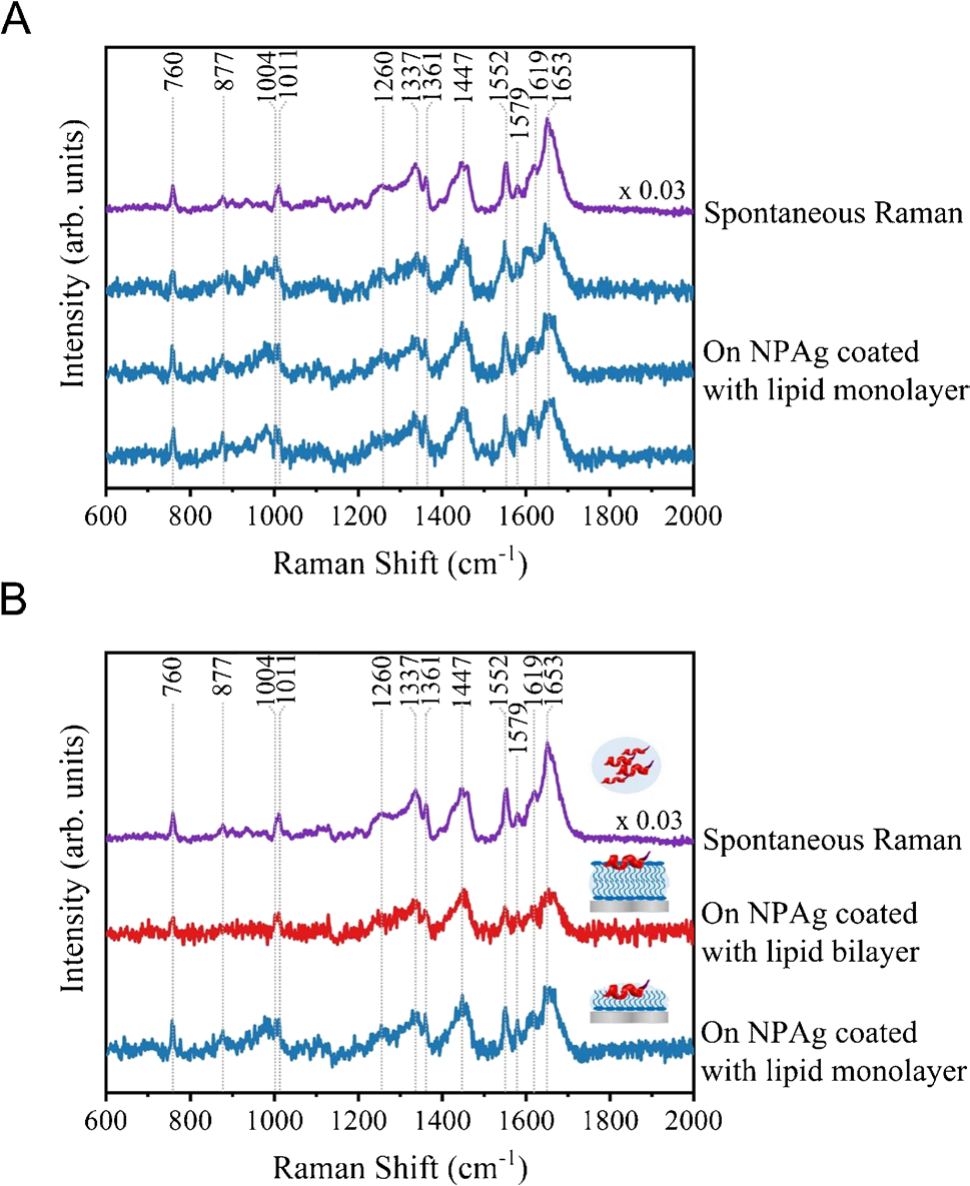
Characterizations of lysozyme in different membrane environments on the lipid-coated NPAg sheets. **A** Spontaneous Raman spectrum of 1 mM lysozyme in solution (purple) and three parallel representative SERS spectra of 1 µM lysozyme on the lipid-coated NPAg sheet with the lipid monolayer coating under cellular surface pressure of 35 mN/m (blue). **B** Spontaneous Raman spectrum of 1 mM lysozyme in solution (purple) and representative SERS spectra of 1 µM lysozyme on the bilayer lipid-coated NPAg sheet (red) and the monolayer lipid-coated NPAg sheet (blue) under cellular pressure of 35 mN/m. (Spontaneous Raman spectra acquisition: 100 mW 514.5 nm laser, 20 × objective, 10 min; SERS spectra acquisition: 5mW 514.5 nm laser, 20 × objective, 30 s)

To examine the feasibility of protein structural characterizations, the SERS spectra of 1 µM lysozyme on the lipid-coated NPAg sheet with the lipid monolayer coating under cellular surface pressure of 35 mN/m (blue) were compared with the spontaneous Raman spectrum of 1 mM lysozyme in solution (purple) in Fig. 3A, showing similar and reproducible vibrational features of lysozyme such as the aromatic residues of Trp (760, 877, 1011, 1337, 1361, 1552, 1579, 1619 cm^−1^), Phe (1004, 1579 cm^−1^), aliphatic residues CH_2_ (1447 cm^−1^), and amide group (1260 and 1653 cm^−1^) [31, 32]. The amide I band at 1653 cm^−1^ and the amide III at 1260 cm^−1^ indicate its α-helical structure, which confirms the preservation of the native conformation of lysozyme on the lipid-coated NPAg sheet. Although lysozyme is known for its bacterial membrane permeability, recent studies reported that lysozyme could not penetrate a phospholipid layer under the cellular lateral pressure at 35 mN/m [33, 34], which is consistent with our observations. That means the compact packing of the lipid monolayer obtained by LB deposition under the cellular surface pressure (35 mN/m) not only provides a biomimetic membrane environment for monitoring protein-membrane interactions, but also acts as a barrier between the proteins and the metallic surface of the NPAg sheet for avoiding protein denaturation. Moreover, the three SERS spectra of lysozyme acquired on different spots of the monolayer lipid-coated NPAg sheet in Fig. 3A are reproducible in spectral features and stable in signal intensities, which further confirm that the compact lipid layer was well-maintained during SERS measurements. Furthermore, the spontaneous Raman spectrum of 1 mM lysozyme on the lipid-coated glass slide (green) and the SERS spectrum of 1 µM lysozyme on the lipid-coated NPAg sheet (blue) were compared in Fig. S5, showing identical spectral features, in particular, the amide I band at 1653 cm^−1^ and the amide III at 1260 cm^−1^. That means lysozyme adopted its native α-helical structure upon interacting with lipid monolayer, which was not affected by the Ag substrate. By contrast, the SERS spectrum of 1 µM lysozyme on the bare NPAg sheet in Fig. S6 presents the distinct peaks at 1336 and 1580 cm^−1^ arising from amorphous carbon [35] and the disappearance of the protein amide I band at 1640 to 1678 cm^−1^ [36], suggesting that part of the protein structures contacted the metal surface to induce severe photothermal damages upon laser excitation without the lipid layer coating. Figure S7 shows the SERS spectra of lysozyme on the lipid-deposited nanoporous silver sheet at 20 different spots, demonstrating the stable spectral features, in particular, the amide I band at 1653 cm^−1^ for the native α-helical structure of lysozyme, which is similar to that of the SERS spectrum of 1 µM lysozyme on lipid-coated nanoporous silver sheet. The absence of the apparent peaks at 1336 and 1580 cm^−1^ implies that lysozyme was well protected by the fully coated lipid layer along the nanoporous silver sheet. Besides, the spontaneous Raman spectrum of 1 mM lysozyme in solution (purple) and representative SERS spectra of 1 µM lysozyme on the bilayer lipid-coated NPAg sheet (red) and the monolayer lipid-coated NPAg sheet (blue) in Fig. 3B show similar spectral features, especially the amide I band at 1653 cm^−1^ and the amide III at 1260 cm^−1^, which indicates that lysozyme adopts the α-helical structure in both the hydrophilic and hydrophobic lipid environments. These results confirm the bio-mimicking lipid monolayer/bilayer environments under the controllable lipid lateral pressure on the lipid-coated NPAg sheets, which also avoids the direct contact between the proteins and the metallic surface on the substrates to preserve native protein structures. Hence, this platform is suitable to characterize the secondary structures of proteins under tunable membrane environments.

**Fig. 4:**
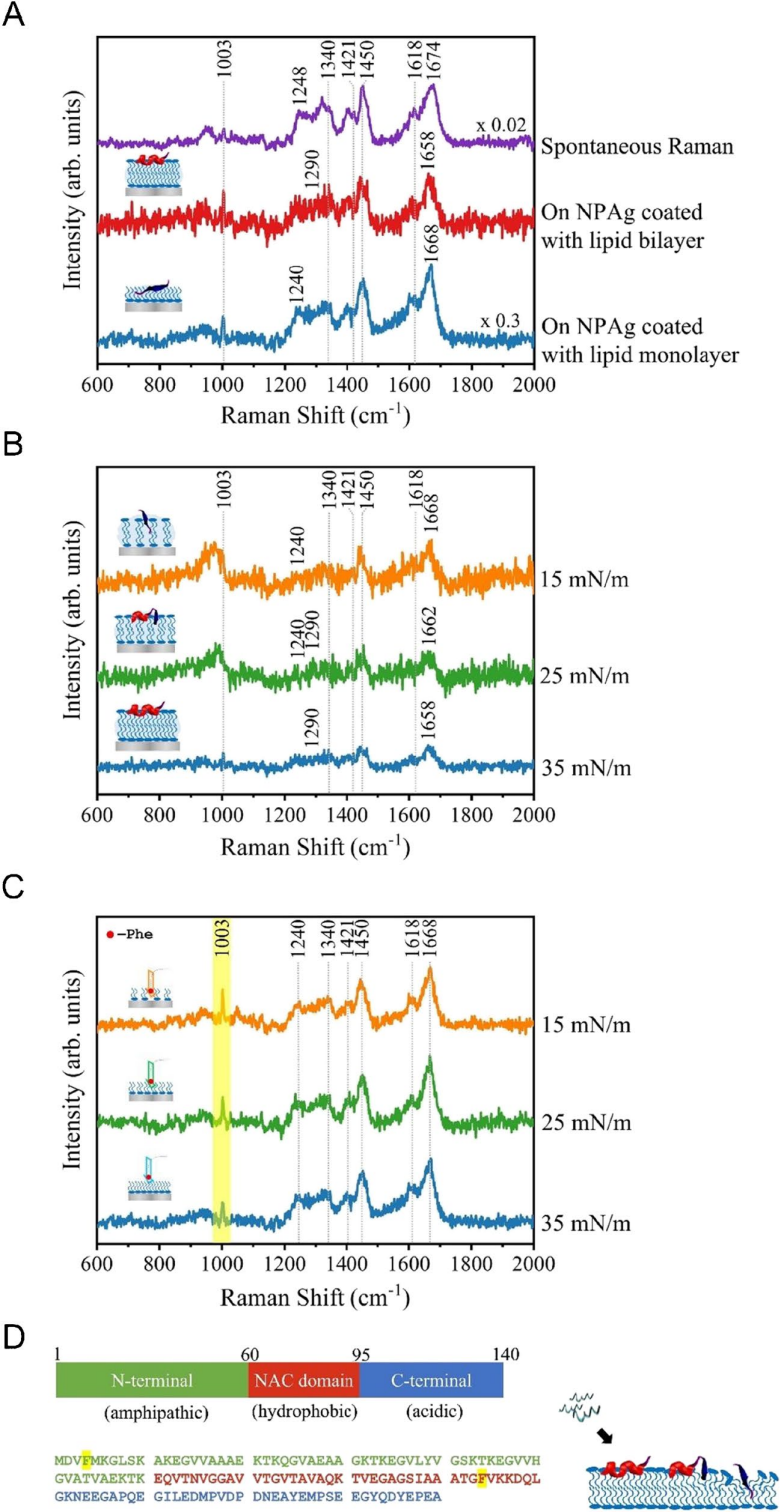
Structural changes of alpha-synuclein in different membrane environments on the lipid-coated NPAg sheets. **A** Spontaneous Raman spectrum of 1 mM alpha-synuclein in solution (purple) and SERS spectra of 1 µM alpha-synuclein on the lipid-coated NPAg sheets with lipid bilayer coating (red) and lipid monolayer coating (blue) under high surface pressure. **B** SERS spectra of 1 µM alpha-synuclein on the lipid-coated NPAg sheets with lipid bilayer coating under the surface pressures of 35 mN/m (blue), 25 mN/m (green) and 15 mN/m (orange). **C** SERS spectra of 1 µM alpha-synuclein on the lipid-coated NPAg sheets with lipid monolayer coating under the surface pressures of 35 mN/m (blue), 25 mN/m (green), and 15 mN/m (orange). (Spontaneous Raman spectra acquisition: 100 mW 514.5 nm laser, 20 × objective, 10 min; SERS spectra acquisition: 5mW 514.5 nm laser, 20 × objective, 30 s.) **D** Sequence of alpha-synuclein (left, the Phe residues are highlighted in yellow) and illustrations of the structural changes of alpha-synuclein upon interacting with different membrane environments (right)

Alpha-synuclein is an intrinsically disordered protein that lacks a fixed secondary structure and adopts heterogeneous conformations in aqueous solution. However, it can alter into certain stable secondary structure upon interaction with different membrane environments. To investigate the disease-related membrane toxicity caused by alpha-synuclein, it was characterized in different membrane environments on the lipid-coated NPAg sheets. Before adding onto the lipid-coated NPAg sheets, the spontaneous Raman spectrum of 1 mM alpha-synuclein aqueous solution was obtained and shown in Fig. 4A (purple). The normal Raman of aqueous alpha-synuclein shows the broad amide I band at 1674 cm^−1^ and the amide III band at 1248 cm^−1^ attributed to its random coil structure, confirming its native form lacks stable secondary structures. Besides, there are several spectral features arising from Phe (1003 cm^−1^), Tyr (1618 cm^−1^), and aliphatic residues CH_2_ (1340, 1421, 1450 cm^−1^) of alpha-synuclein [37]. In comparison, the SERS spectra of 1 µM alpha-synuclein on the lipid-coated NPAg sheets with lipid bilayer coating (Fig. 4A, red) and lipid monolayer coating (Fig. 4A, blue) also demonstrate these characteristic vibrations of alpha-synuclein, which confirms the detection of alpha-synuclein at its physiological concentration of 1 µM in membrane environments, which concentration is below the detection limit of spontaneous Raman. Interestingly, the SERS spectrum of alpha-synuclein on the lipid bilayer-coated NPAg sheet in Fig. 4A (red) shows the peaks of 1658 cm^−1^ in the amide I region and 1290 cm^−1^ in the amide III region, which indicates the α-helical structure of alpha-synuclein upon interaction with hydrophilic lipid heads, corresponding to previous studies [4, 38]. However, the SERS spectrum of alpha-synuclein on the lipid monolayer-coated NPAg sheet in Fig. 4A (blue) exhibits the amide I band of 1668 cm^−1^ and the amide III band of 1240 cm^−1^, which are assigned to the β-sheet structure of alpha-synuclein. This observation indicates a different structural transition of alpha-synuclein when exposed to hydrophobic lipid tails. Overall, the distinct structures of alpha-synuclein upon the interactions with different membrane regions are characterized on the lipid-coated NPAg sheets. It is revealed that the hydrophobic membrane interior drives alpha-synuclein to adopt the β-sheet structure, while the hydrophilic membrane surface induces the α-helical structural conversion, which originated from its random coil structure in water. More importantly, the lipid monolayer/bilayer coated NPAg sheets provide significant enhancement that allows direct detection of alpha-synuclein at physiological concentrations that are not accessible with spontaneous normal Raman.

Considering the β-sheet-containing alpha-synuclein species are responsible for further membrane disruption, it is crucial to monitor its structural conversions under well-defined membrane environments [39]. To control the exposure of membrane interior, the packing density of the lipid bilayer coating was adjusted to cellular pressure of 35 mN/m, solid phase pressure of 25 mN/m, and liquid phase pressure of 15 mN/m during LB deposition. Figure 4B presents the SERS spectra of 1 µM alpha-synuclein on the lipid-coated NPAg sheets with lipid bilayer coating under the different surface pressures in LB depositions. Compactly packed under high cellular surface pressure of 35 mN/m, the lipid heads on the membrane surface stabilized the α-helical alpha-synuclein, as indicated by the characteristic peak of 1658 cm^−1^ in the amide I region in Fig. 4B (blue). However, the amide I peak shifts from 1658 to 1668 cm^−1^ under the liquid phase of the lipid bilayer at the surface pressure of 15 mN/m, which is further verified by the emerging peak at 1668 cm^−1^ in the difference spectra of 1 µM alpha-synuclein on the lipid-coated NPAg sheets with lipid bilayer coating under the surface pressure of 35 mN/m, 25 mN/m, and 15 mN/m in Fig. S8. With the decreasing surface pressure, an increasing amount of the membrane interior area was exposed to alter the secondary structure of alpha-synuclein into β-sheet. That means the β-sheet formation of alpha-synuclein could be driven by the local hydrophobic environment from the exposed lipid tails. To further explore the interactions between alpha-synuclein and the interior lipid tails for membrane insertion, alpha-synuclein was characterized on the lipid monolayer-coated NPAg sheets with different packing densities under the controllable LB deposition pressures. As shown in Fig. 4C, the sharp amide I band at 1668 cm^−1^ and the amide III band at 1240 cm^−1^ are attributed to the β-sheet structure of alpha-synuclein. Interestingly, the peak intensities of 1003 cm^−1^ (Phe) increase with the decreasing lipid densities (LB deposition pressures), indicating the stronger SERS enhancement from the deeper insertion of the Phe residues at the looser lipid packings. According to the sequence of alpha-synuclein (Fig. 4D), one Phe residue locates in the N-terminal region (Phe-4) and the other Phe sits in the hydrophobic non-amyloid-β component fragment (Phe-94). It is proposed that Phe-4 and Phe-94 might both contribute to this signal change since previous studies have identified that the first 14 residues of alpha-synuclein N-terminal (including Phe-4) enable membrane insertion [40] and the β-structure core region of alpha-synuclein protofibrils (near Phe-94) is immersed inside membranes [41–43]. These results confirm the assumption that the protein lipophilic elements could promote membrane insertion through strong hydrophobic interactions [39, 44].

Because it is challenging to characterize the dynamic structural conversions of alpha-synuclein, the molecular mechanisms of alpha-synuclein misfolding and aggregation for membrane toxicity still remain unclear. There are two proposed models to illustrate the structural features of alpha-synuclein in membrane disruptions. The toroidal model involves the binding of alpha-synuclein monomers onto the membrane surface with the α-helical structure to form pores or channels in membranes [45], whereas the barrel model incorporates the β-sheet-rich alpha-synuclein oligomers with central pores for membrane permeability [46, 47]. Our results indicate that the hydrophobic lipid environment inside membranes drives alpha-synuclein to adopt the β-sheet structure alternative to the α-helical structure on the hydrophilic membrane surface, which originated from its disorder monomers in aqueous solution (Fig. 4D). Since lipid tails are unavoidable during membrane insertions, the hydrophobic interactions with the submerged protein residues play an important role in determining the folded states and early aggregates of alpha-synuclein. It is supported by the previous study that the α-helical alpha-synuclein is settled in the vicinity of lipid heads on the membrane surface [48], while the hydrophobic residues are buried in the interior of membranes [49]. The observation of the β-sheet containing alpha-synuclein upon interacting with the hydrophobic lipid regions might be linked to the β-sheet-rich oligomers responsible for membrane disruptions and the early aggregates prior to mature fibrillation [2, 3, 39]. Therefore, owing to the convenient tunability in preparing different lipid monolayer/bilayer packing pressures, the lipid-coated NPAg sheets enable the characterizations of alpha-synuclein in different controllable membrane environments, which reveals the β-sheet formation of alpha-synuclein driven by the interactions with the hydrophobic lipid tails in addition to its α-helical conversion upon binding to the hydrophilic lipid heads. The lipid monolayer/bilayer-coated NPAg platform can be utilized in the future investigation of protein-membrane interaction with good tunability and sensibility to track protein changes under low physiological concentration by SERS enhancement.

## Conclusions

Overall, we presented a sensitive and convenient SERS platform to provide tunable lipid monolayer/bilayer environments and investigate protein secondary structures in protein-membrane interactions under low physiological concentrations. Prepared by the pressure-controlled LB transfer, the lipid-coated NPAg sheets empower in situ characterizations of proteins under hydrophilic and hydrophobic environments, which preserve the native protein structures while enhancing their SERS signals from the underlying metallic nanostructured bases. More importantly, it enables the fine-tuning of the lipid monolayer/bilayer packing density to monitor the protein structures upon interacting with the hydrophilic lipid heads and the hydrophobic lipid tails, respectively. In different lipid environments, lysozyme maintained the same α-helical structure. Interestingly, alpha-synuclein folded into the α-helical structure on the negatively charged lipid heads, while the exposure of the hydrophobic lipid tails induced the β-sheet formation of alpha-synuclein in both monolayer lipid tails and low-pressure liquid phase lipid bilayer, which originated from its unstructured monomers in aqueous. The observation of the β-sheet-containing alpha-synuclein in hydrophobic membrane regions is different from its well-known membrane-bound α-helical structure, which might provide important clues to understand the membrane toxicity and the pathogenic aggregation mechanism of alpha-synuclein linked to Parkinson’s disease. With great tunability on lipid monolayer/bilayer packing and good spectral detection sensitivity allowing physiological concentrations, our lipid-coated NPAg SERS platform opens a new door to study protein-membrane interactions in various membrane environments that provide valuable insights into a variety of crucial membrane-related biological processes.

## Supplementary Information

Below is the link to the electronic supplementary material.

Supplementary file1 (PDF 2555 KB)
