# Supplementary material for: Tunable lipid-coated nanoporous silver sheet for characterization of protein-membrane interactions by surface-enhanced Raman scattering (SERS)
Source: Anal Bioanal Chem. 2023 Apr 21;415(16):3243–53. doi: 10.1007/s00216-023-04701-y (PMC10287797; doi:10.1007/s00216-023-04701-y)
Supplement: Supplementary file 1 — Supplementary file1 (PDF 2555 KB) [file 216_2023_4701_MOESM1_ESM.pdf]

Electronic Supplementary Material

Tunable lipid coated nanoporous silver sheet for characterization of protein-membrane interactions by surface-enhanced Raman scattering (SERS)

Hongni Zhu [a, b], Jianing Zhang [a, b], Xin Dai [b, c], Vince St. Dollente Mesias [b], Huanyu Chi [a, b], Congcheng Wang [d], Chi Shun Yeung [b, e], Qing Chen [b, d], Wei Liu [*, c], and Jinqing Huang [*, a, b]

[a.] *HKUST-Shenzhen Research Institute, No. 9 Yuexing first RD, Hi-tech Park, Nanshan, Shenzhen 518057, China*

[b.] *Department of Chemistry, The Hong Kong University of Science and Technology, Clear Water Bay, Kowloon, Hong Kong, China*

[c.] *Department of Chemistry, The University of Hong Kong, Pokfulam Road, Hong Kong, China*

[d.] *Department of Mechanical and Aerospace Engineering, The Hong Kong University of Science and Technology, Clear Water Bay, Kowloon, Hong Kong, China*

[e.] *Department of Civil & Environmental Engineering, The Hong Kong Polytechnic University, Hong Kong, China*

*Corresponding Author:

Jinqing Huang

Email: jqhuang@ust.hk

Wei Liu

Email: wliu276@hku.hk

Table of Contents

Fig. S1 Contact angles of water on the monolayer lipid-coated NPAg sheets under the surface pressure of 15 mN/m, 25 mN/m, and 35 mN/m (top) and the bilayer lipid-coated NPAg sheets under the surface pressure of 15 mN/m, 25 mN/m, and 35 mN/m (bottom). 3

Fig. S2 BET analysis of nanoporous silver sheet 3

Fig. S3 Raman spectra of the lipid monolayer coated on the glass slide (blue) and the lipid monolayer coated on the Ag substrate (green).. 4

Fig. S4 SERS spectra of lipid-coated nanoporous silver sheet at 100 different spots. 4

Fig. S5 Spontaneous Raman spectrum of 1 mM lysozyme on the lipid-coated glass slide (green) and SERS spectrum of 1 μM lysozyme on the lipid-coated NPAg sheet (blue). 5

Fig. S6 SERS spectrum of 1 μM lysozyme on bare nanoporous silver sheet (green) and lipid-coated nanoporous silver sheet (blue). 5

Fig. S7 SERS spectra of lysozyme on lipid-coated nanoporous silver sheet at 20 different spots. 6

Fig. S8 SERS spectra and difference spectra of 1 μM alpha-synuclein on the lipid-deposited NPAg sheets with lipid bilayer coating under the surface pressures of 35 mN/m, 25 mN/m, and 15 mN/m, respectively. 6

Table S1 Main peak assignment of lysozyme.. 7

Table S2 Main peak assignment of alpha-synuclein 7

References 8

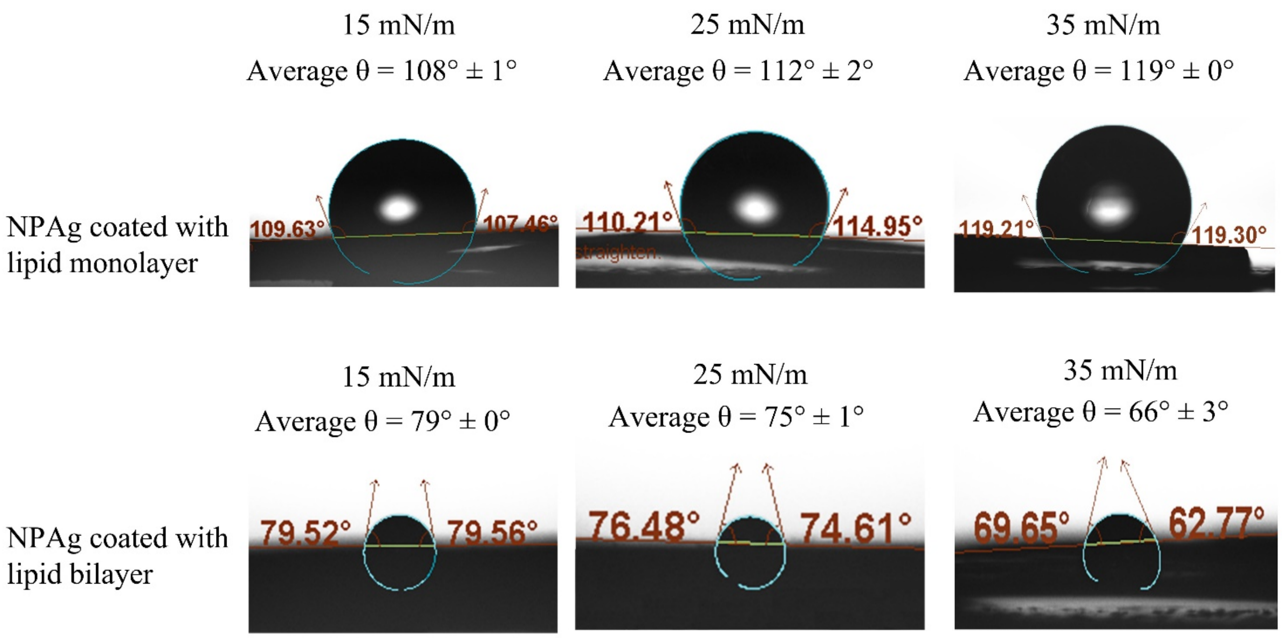

Fig. S1 Contact angles of water on the monolayer lipid-coated NPAg sheets under the surface pressure of 15 mN/m, 25 mN/m, and 35 mN/m (top) and the bilayer lipid-coated NPAg sheets under the surface pressure of 15 mN/m, 25 mN/m, and 35 mN/m (bottom).

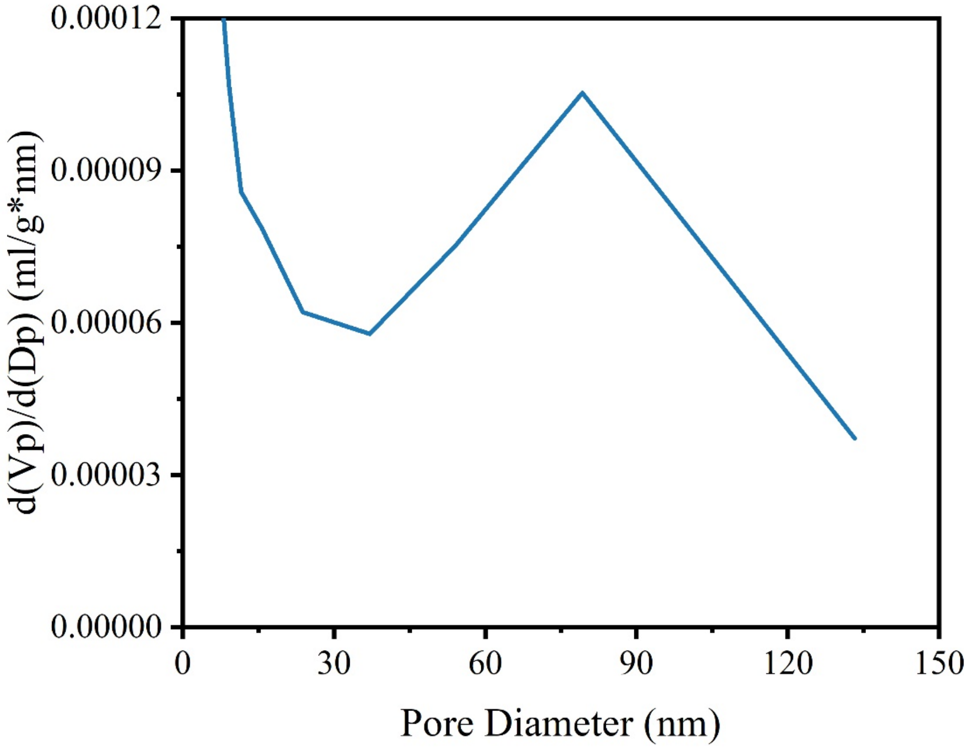

Fig. S2 BET analysis of nanoporous silver sheet

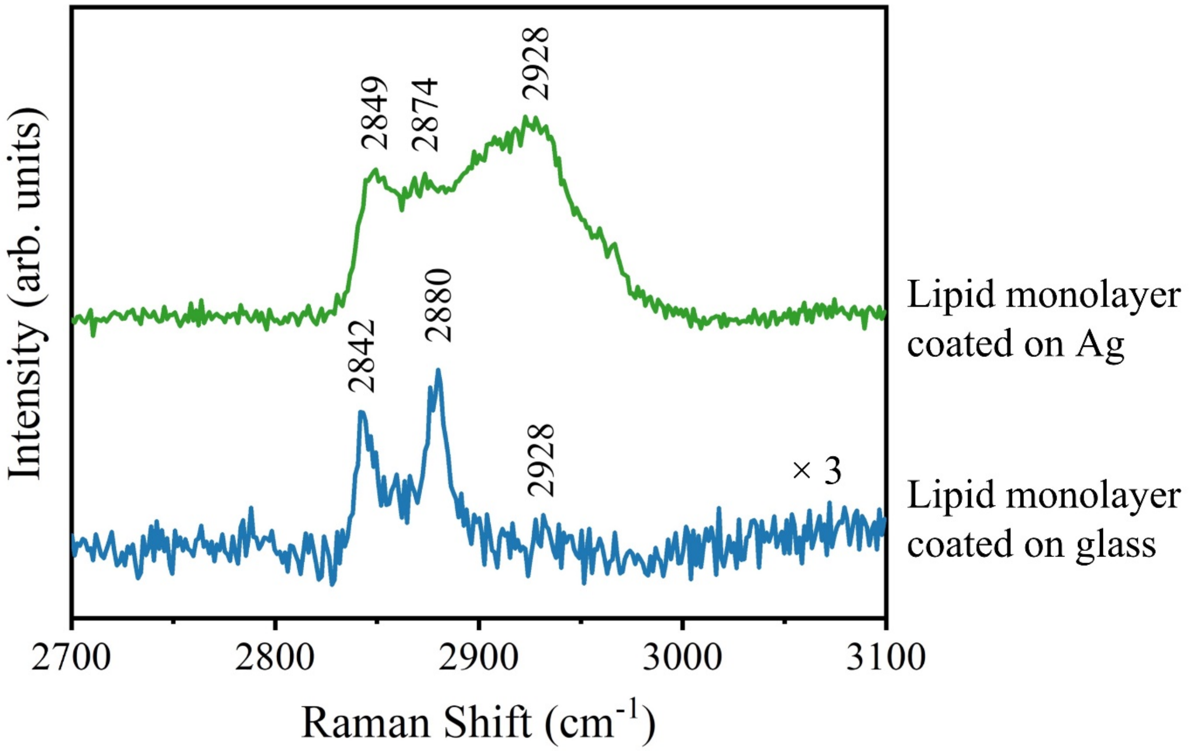

Fig. S3 Raman spectra of the lipid monolayer coated on the glass slide (blue) and the lipid monolayer coated on the Ag substrate (green).

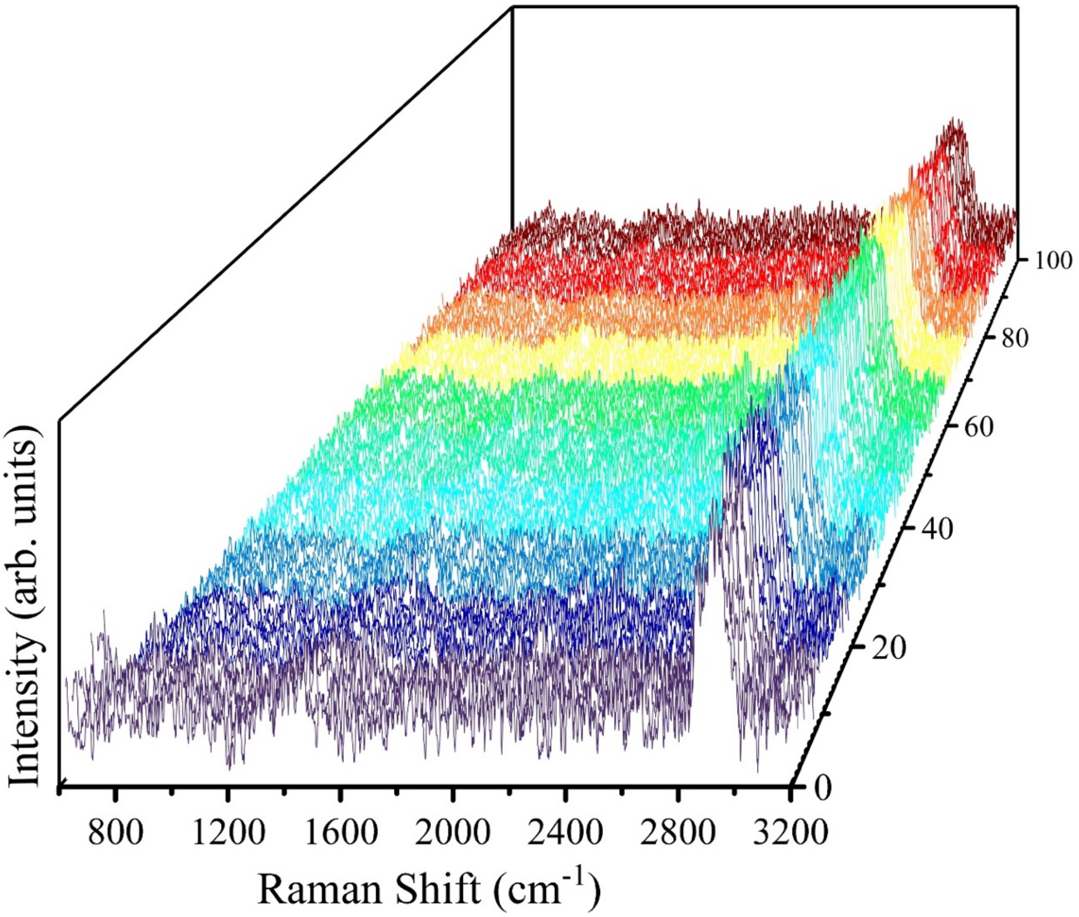

Fig. S4 SERS spectra of lipid-coated nanoporous silver sheet at 100 different spots.

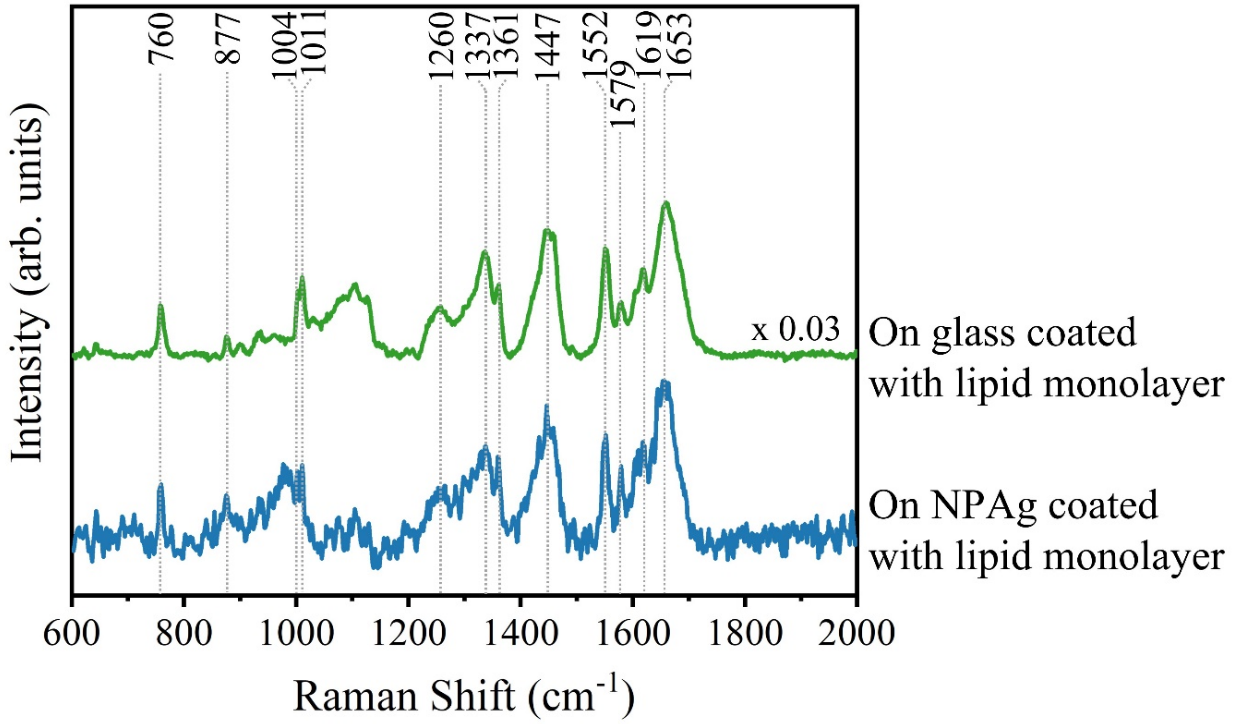

Fig. S5 Spontaneous Raman spectrum of 1 mM lysozyme on the lipid-coated glass slide (green) and SERS spectrum of 1 µM lysozyme on the lipid-coated NPAg sheet (blue).

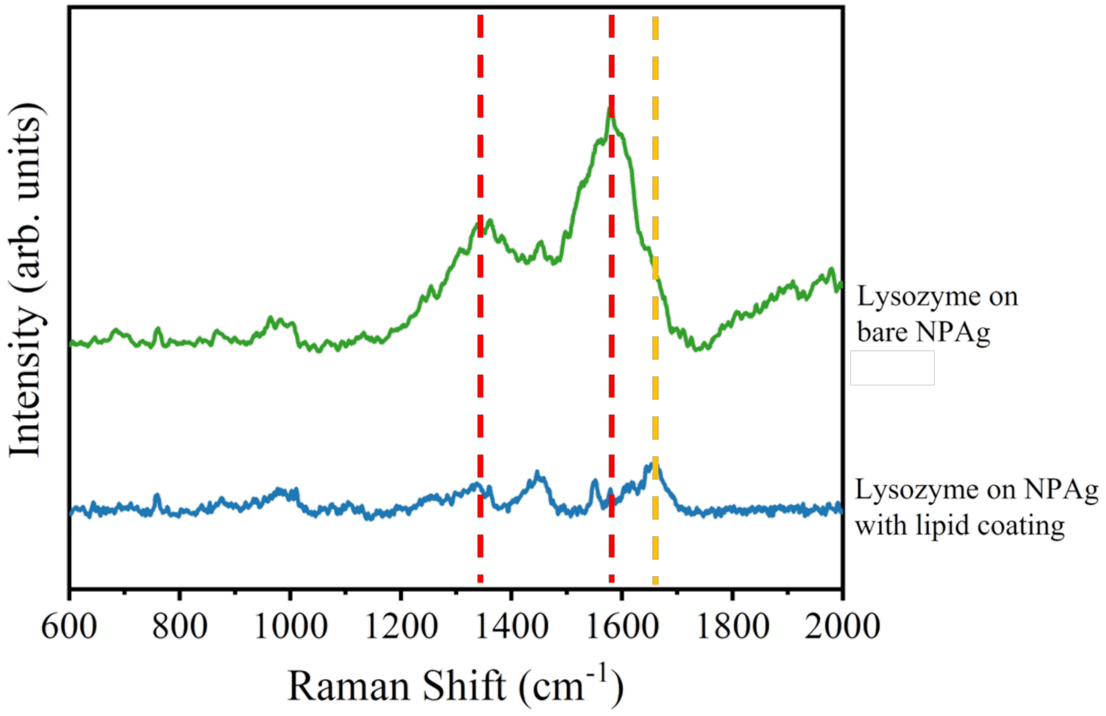

Fig. S6 SERS spectrum of 1 µM lysozyme on bare nanoporous silver sheet (green) and lipid-coated nanoporous silver sheet (blue).

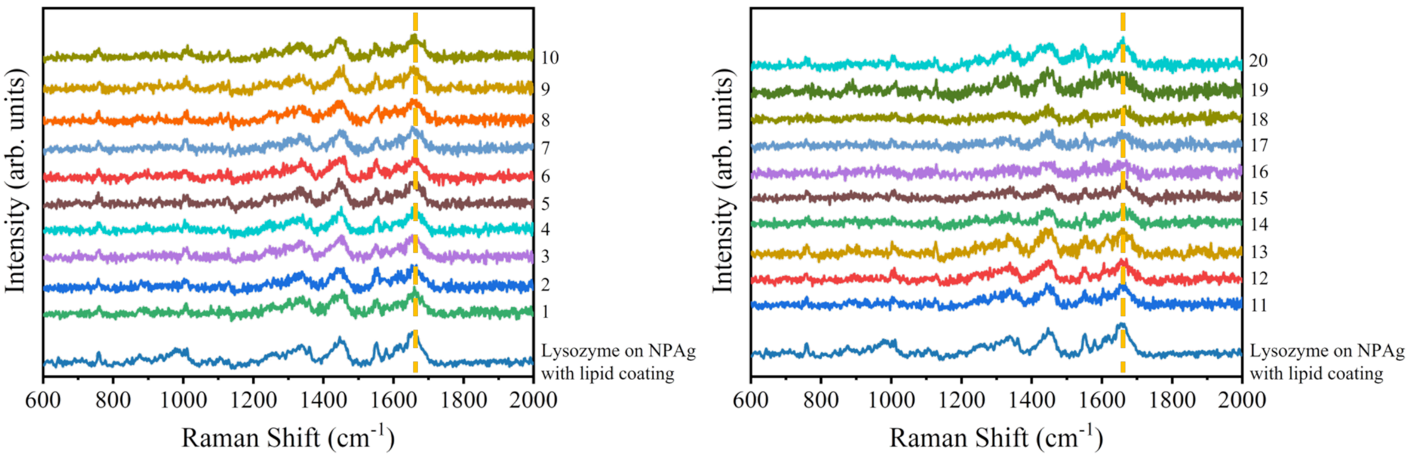

Fig. S7 SERS spectra of lysozyme on lipid-coated nanoporous silver sheet at 20 different spots.

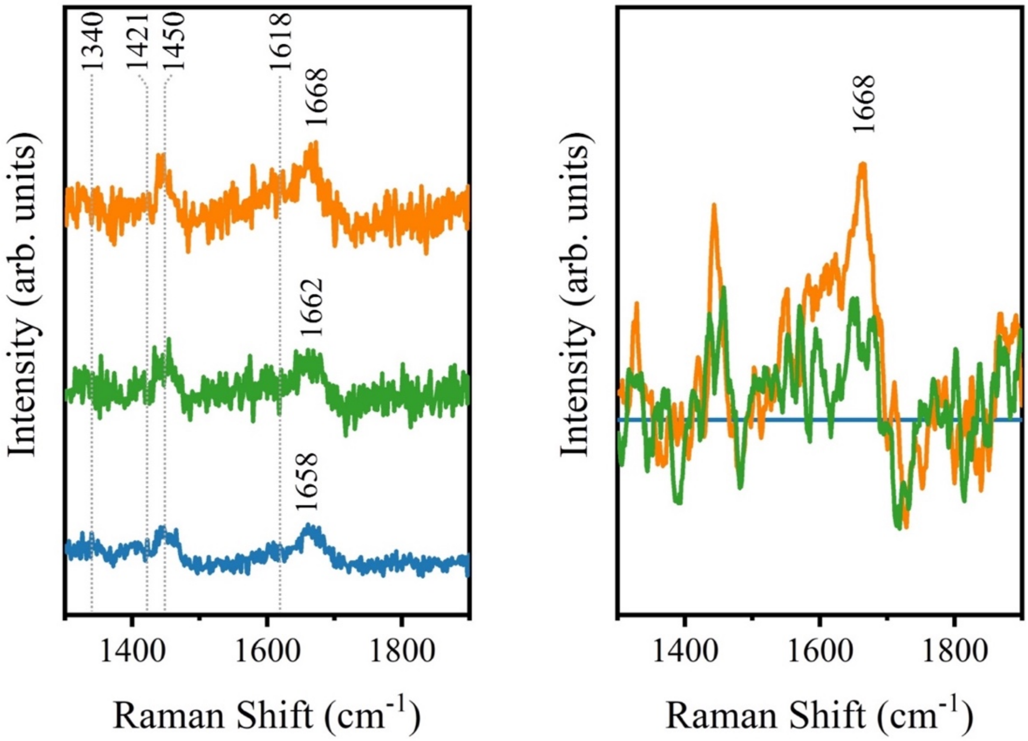

Fig. S8 SERS spectra and difference spectra of 1 μM alpha-synuclein on the lipid-deposited NPAg sheets with lipid bilayer coating under the surface pressures of 35 mN/m, 25 mN/m, and 15 mN/m, respectively.

Table S1 Main peak assignment of lysozyme [1, 2]

**Raman Shift (cm^{-1})**	**Tentative Assignment**
**760**	Trp
**877**	Trp
**1004**	Phe
**1011**	Trp
**1260**	Amide III
**1337**	Trp
**1361**	Trp
**1447**	C-H bending
**1552**	Trp
**1579**	Trp/Phe
**1619**	Trp
**1653**	Amide I

Table S2 Main peak assignment of alpha-synuclein [3]

**Raman Shift (cm^{-1})**	**Tentative Assignment**
**1003**	Phe
**1240**	Amide III (β-sheet)
**1248**	Amide III (random coil)
**1290**	Amide III (α-helical)
**1340**	CH_2 deformation
**1421**	CH_2, CH_3 deformation
**1450**	CH_2, CH_3 deformation
**1618**	Tyr
**1658**	Amide I (α-helical)
**1668**	Amide I (β-sheet)
**1674**	Amide I (random coil)

References

[1] Xu LJ, Zong C, Zheng XS, Hu P, Feng JM, Ren B. Label-Free Detection of Native Proteins by Surface-Enhanced Raman Spectroscopy Using Iodide-Modified Nanoparticles. Anal Chem. 2014;86(4):2238-45.

[2] Hu J, Sheng RS, Xu ZS, Zeng Y. Surface enhanced Raman spectroscopy of lysozyme. Spectrochim Acta A Mol Biomol Spectrosc. 1995;51(6):1087-96.

[3] Maiti NC, Apetri MM, Zagorski MG, Carey PR, Anderson VE. Raman Spectroscopic Characterization of Secondary Structure in Natively Unfolded Proteins: α-Synuclein. J Am Chem Soc. 2004;126(8):2399-408.
